# Determining minimum number of valid days for accurate estimation of sedentary behaviour and awake-time movement behaviours using the ActivPAL3 in nursing home residents

**DOI:** 10.1186/s11556-023-00329-0

**Published:** 2023-10-07

**Authors:** Pau Farrés-Godayol, Miguel Ángel Ruiz-Díaz, Philippa Dall, Dawn A. Skelton, Eduard Minobes-Molina, Javier Jerez-Roig, Maria Giné-Garriga

**Affiliations:** 1 Research group on Methodology, Methods, Models and Outcomes of Health and Social Sciences, Faculty of Health Sciences and Welfare, Centre for Health and Social Care Research (CESS), University of Vic-Central University of Catalonia (UVic-UCC), C. Sagrada Família, 7, Vic, 08500 Spain; 2 Institute for Research and Innovation in Life Sciences and Health in Central Catalonia (IRIS-CC), Vic, Spain; 3 Department of Social Psychology and Methodology, Psychology Faculty, Universidad Autónoma de Madrid, Madrid, Spain; 4 Research Centre for Health (ReaCH), School of Health and Life Sciences, Glasgow Caledonian University, Glasgow, UK; 5 Blanquerna Faculty of Psychology, Education and Sport Sciences, Ramon Llull University, Barcelona, Spain; 6 Blanquerna Faculty of Health Sciences, Ramon Llull University, Barcelona, Spain

**Keywords:** Nursing home residents, Older adults, Sedentary behaviour, activPAL3, Minimum wear time duration

## Abstract

**Introduction:**

Scarce evidence is available about the minimum number of valid days wearing the activPAL3 to obtain a precise estimate of sedentary behaviour (SB) and awake-time movement behaviours (ATMB) in nursing home (NH) residents. The study aimed to determine the minimum number of valid days required for accurately estimate SB and ATMB using the activPAL3 device in NH residents. It also investigated how the starting point of a day (the 24-h period) impacted reliability.

**Methods:**

Participants wore an activPAL3 for 7 consecutive days. The data was classified in two-time blocks (00:00 Ante Meridiem (AM)—00:00 AM midnight vs 12:00 Post Meridiam (PM) -12:00 PM midday) and the sample was stratified into two groups according to their capacity to stand and walk, to examine if timing of sampling or physical functioning affected minimum wear time. SB, ATMB, sociodemographic, and health-related variables were collected. Sensitivity of the time-blocks were tested through the dispersion frequencies and differences between blocks through Kolmogorov–Smirnov test for normality; parametric variables through two-related means T-test and Wilcoxon test for non-parametric data. Reliability was assessed with the Cronbach's Alpha and the intra-class correlation coefficient (ICC), using a one-factor model estimating the reliability for each measurement day loading in the same latent factor.

**Results:**

Ninety-five NH residents (81.1% women; age = 85.8 ± 7.2 years) were included. The midnight block had higher reliability, sensitivity and no statistically significant differences between days were found. At least three consecutive days of monitoring were necessary to achieve a reliability of ICC ≥ 0.8 for those NH residents able to stand and walk and six days for those unable.

**Conclusions:**

NH residents who are able to stand and walk require a minimum of three consecutive days wearing the device, while those who are unable require at least six days due to their highly homogenous daily routines and sensitivity to PA events. Regardless of the activPAL3 recording start time, data processing should reference the midnight time block.

## Introduction

The current predictions for Europe's demographic trends show that in 2050, the demographic pyramid will be heavily aged; health and social expenditure will represent a challenge for all the governments and societies worldwide to support older adults’ care needs [1]. One modifiable health-related risk factor with consequences that are associated with an increase in healthcare expenditure is sedentary behaviour (SB) [2]. SB can be defined as any waking behaviour characterized by an energy expenditure ≤ 1.5 Metabolic Equivalent Task (MET) while in a sitting, reclining or lying posture [3]. In recent decades, SB has gained popularity as a risk factor for various health-related conditions such as cardiovascular diseases, hypertension, cancer, metabolic disorders like type 2 diabetes, dyslipidemia, and all-cause mortality [4–7].

It is important to distinguish between prolonged episodes of SB and short episodes of interrupted SB by brief episodes of physical activity (PA) or postural changes [8]. Although evidence is limited, results indicate that breaking up prolonged SB episodes offers cardiometabolic benefits [9–12]. On the other hand, for uninterrupted SB episodes, each additional hour of SB per day increases overall mortality risk by 2%, and by 5% for those spending over 7 h a day in SB, regardless of how much PA is undertaken [7]. However, while for some health outcomes the effect of moderate PA appears to be independent of SB, moderate PA can modify the harmful effects of prolonged SB, through factors like level, duration, intensity, or time spent in PA [13]. For example, approximately 60 to 75 min of moderate PA per day are needed to offset the adverse health effects of prolonged SB episodes [14, 15].

This is significant in the context of aging, as PA levels decline and SB increases with age, creating a vicious cycle where the physical capacity loss induced by SB and physical inactivity leads to more SB and increased mortality risk [16, 17]. Older individuals experience more functional limitations due to chronic diseases or multimorbidity situations than the rest of the population, leading to the accumulation of prolonged and uninterrupted SB episodes [13, 18–21]. Among the entire population, older adults are the most sedentary, and as age increases, the accumulation of time spent in SB gradually rises, while the time spent in weight-bearing activities proportionally decreases [22, 23]. Moreover, in older individuals, there is evidence linking high levels of SB with an accelerated aging process, frailty, urinary incontinence, musculoskeletal disorders like osteoporosis, and mental disorders like dementia, depression, and anxiety [24–29].

However, within older populations, nursing home (NH) residents are the least active and accumulate the highest percentage of prolonged and uninterrupted SB bouts compared to community-dwelling seniors [22–24, 30–34]. The daily activity of a NH resident consists of spending between 71 and 98% of their daytime in SB, accumulated in uninterrupted periods of approximately 60 min based on their level of dependency, 20% engaged in light-intensity PA typically related to self-care activities, eating, or mobility, and the remaining 1% in moderate to vigorous-intensity PA (MVPA) [31, 35–37].

To assess SB in older adults, objective methods, such as accelerometers, and subjective methods, like self-reported questionnaires, are used. However, self-reported questionnaires on SB time among older adults exhibit an average underestimation of 4.6 h per day. When compared to objective methods, subjective methods are inaccurate and unreliable in assessing SB among the older adults [38–40].For these reasons, there has been a growing interest in validating activity devices to objectively measure PA and SB in this population [40, 41]. One of the most used devices and considered the gold standard to assess SB in different populations is the activPAL (Pal Technologies, UK). The activPAL collects accelerometer-derived information about thigh inclination that is highly accurate in identifying lying, sitting, and upright positions and has been validated in laboratory and in free living conditions in older adults [42–44]. According to the literature, the recommendations to use the activPAL in older adults are to employ a 24-h wearing protocol for at least 7 days [45]. However, scarce evidence is currently available about the minimum number of valid days wearing the activPAL necessary to obtain an accurate estimate of SB, and when the device must start recording in NH residents to obtain reliable data of their SB and awake-time movement behaviours (ATMB) [33].

Furthermore, one of the determinants for methodological reliability on recorded data with the activPAL3 relies on the compliance in wearing the device continuously for several days by the NH population. Wearing devices for many days could be difficult for those residents with cognitive impairment, because they might tend to forget why they are wearing the device and may take it off [46]. Previous evidence suggests that NH employees in Catalonia work in understaffed and overworked conditions. Being actively included in a research study and tasked with monitoring the devices could add to their already high daily workload and contribute to burnout, potentially leading to the loss of devices. Additionally, the device's attachment to the resident's skin may cause problems to more vulnerable skin, irritations or allergies, which the NH staff would have to manage [47].

One processing decision that is required for activity monitor data acquired using a continuous 24-h wear protocol, is to define the day to be used for analysis [45]. Many studies use a calendar day (24 h from the fixed time of midnight), but an alternative is to select a person-oriented day (e.g., from time of waking until the time of waking the next day). The person-centred approach reflects the actual behaviour of the participant, but this can introduce additional inter participant variability as time between subsequent wake times may not be exactly 24 h [45]. Pragmatic aspects of study design, such as staff availability, may influence the selection of a start time for fixed 24-h periods for analysis. For example, a study where the monitor was attached in a morning and worn continuously until the afternoon of the same day the following week, would be able to analyse seven 24-h periods starting at midday, but only six 24-h periods starting at midnight. It is currently not known whether there is a statistical difference in reporting of ATMB and SB when using different starting times of a day as the unit of analysis.

Therefore, the aims of the current study were to determine the number of valid days to obtain an accurate estimate of SB and the ATMB using the activPAL3 monitor and to explore whether the start point of a day (24-h period) influenced reliability in NH residents.

## Materials and methods

### Design

Data were collected in a cross-sectional study in five NHs of Osona county (Central Catalonia, Spain), from January 2020 until March 2020 (when data collection had to stop due to the COVID-19 pandemic). This is a validation study which used the baseline data from the OsoNaH Project that aimed to evaluate the association between urinary incontinence and SB among NH residents [48], registered in Clinical Trials (NCT04297904) and approved by the Ethics and Research Committee of the University of Vic – Central University of Catalonia (reference number 92/2019).

### Participants

We included NH residents (males and females) aged 65 years or older and who lived in the permanently in the institutions. Individuals in a coma, palliative care, (prognosis of short life), hospitalised were excluded. Signed, informed, consent was received either from the resident themselves or their legal guardian.

### Procedures

The initial contact with the NH involved email and phone communication to explain the project and address inquiries. Subsequently, interested NH directors received the information sheet and consent forms. Consent was formally obtained from participating NH directors. Afterward, resident lists were obtained, and individuals were chosen based on inclusion/exclusion criteria. Using IBM SPSS Statistics software (2021 Version 28.0. IBM Corp.: Armonk, NY, USA)., a randomization process was conducted, and selected residents or their legal guardians were informed and provided informed consent upon agreement. NH staff who agreed also signed informed consent. Participants were informed of their right to interrupt or discontinue assessments due to fatigue, as well as the option to withdraw from the study without explanation. The research team underwent training, following standardized procedures, with inter-rater reliability assessed using Kappa and interclass correlation coefficient (ICC) on data from 20 residents. ICC scores exceeded 0.75 for all physical tests. The results for these 20 residents were excluded from the final study sample. Following reliability calibration, a pilot study was conducted with a distinct sample of 36 residents, whose data were included in the analysis.

Sociodemographic and health related variables such as age and sex, were obtained from the NH records and checked with the NH staff. Functional status was measured using the modified Barthel Index by Shah et al. without the continence items [49]. Continence status was assessed using Section H of Minimum Data Set version 3.0 [50]. Physical performance was examined using the Short Performance Physical Battery (SPPB) and finally, cognitive status was assessed using the Pfeiffer Scale [51, 52]. The NH residents sample were stratified in 2 groups according to whether they were able or unable to stand and walk. The classification of the groups was based on the answers from the NH staff when the team asked them about residents capabilities on standing and walking (those able to stand and walk with technical support such as walkers, braces, and crutches were considered to be capable), and confirmed by the SPPB. Those classified by the NH staff as unable to stand and walk and with a SPPB score of 0, were assigned to the unable group and those considered able by the NH staff were assigned to the able group.

The participants wore an activPAL3 activity monitor (PAL Technologies Ltd., Glasgow, UK) size: 53 × 35 × 7 mm; weight: 15g, with a triaxial accelerometer sampling at 20 Hz with a range of ± 2 g. The activPAL is considered the gold standard to assess SB and has been validated in laboratory and in free living conditions in older adults [42–44]. The device was worn on the anterior medial part of the right thigh. The device was sealed with a flexible nitrile cover and adhered to the skin with a hypoallergenic adhesive dressing (TegadermTM Roll, 3MTM) to provide waterproof protection, which allowed the participants to wear the device continuously without removal for showering or sleeping. The monitor was programmed to start recording at a later time after the research appointment (usually 12:00 PM) and set to record for 7 days. The device captured the data continuously following the 24 h protocol during both awake and sleeping time, for 7 consecutive days and then was removed by the researcher.

### Accelerometer data processing

ActivPAL3 data were downloaded from the device to a laptop using the manufacturer’s software (PALconnect, V8.12.6.118, PAL Technologies Ltd., Glasgow, UK). Data were inspected by two experts using the visual tools in the manufacturer’s software (PALanalysis, V8.11.8.74, PAL Technologies Ltd., Glasgow, UK) to identify potential non-wear, data loss from monitor malfunction or the battery stopping early. The data and time where monitor wear/data collection stopped and the reasons for any data loss were recorded for each participant. Time in bed was initially identified using the automated algorithm in the manufacturer’s software (the CREA algorithm). The automated selection was then inspected visually by two researchers, and wake and bed times were adjusted through visual identification from the posture data represented on the time in bed adjustments section (e.g., sitting/lying, standing or stepping).

Data were exported for the activPAL software in the event format (continuous periods of a single activity) categorised using the original algorithm (VANE algorithm; data categorised as sit/lie, stand or step). Additional data processing was conducted using a custom excel macro (known as the HSC analysis program, developed by Dr Philippa Dall (co-author) and Professor Malcolm Granat, School of Health and Life Sciences, Glasgow Caledonian University) [53]. For each participant, individual step events were aggregated into walking events, and upright events were created as an aggregate of contiguous standing and walking events. The wake and bed times were used to isolate waking activity (events were split at the wake/bed time). Data was then organised into two different definitions of days (time-blocks) for analysis. Each time-block consisted of 24-h periods: the midnight time block from 00:00 AM to 00:00 AM and midday time block from 12:00 PM to 12:00 PM. Events were split at the start/end times of each day. Participants wore the monitor on eight calendar days, putting it on in the morning of day 1 and taking it off in the afternoon of day 8. When the monitor was worn per protocol, this allowed analysis of six days in the midnight time block (from day 2 until day 7), and seven days in the midday time block (from 12:00 PM on day 1 until 12:00 PM on day 8).

### Outcome measures

Outcome measures were calculated for each day in each time-block. The SB variables extracted were: 1) absolute time in SB in hours, 2) % of time awake in SB, 3) number of SB bouts < 30 min, 4) absolute time spent in bouts < 30 min, 5) % of time awake in bouts < 30 min, 6) number of SB bouts between 30–60 min, 7) absolute time spent in bouts between 30–60 min, 8) % of time awake in bouts between 30–60 min, 9) number of SB bouts > 60 min, 10) absolute time spent in bouts > 60 min, 11) % of time awake in bouts > 60 min and 12) average duration of SB bouts in minutes. The awake time movement behaviour (ATMB) extracted variables were: 13) hours awake, 14) standing duration in hours, 15) % of time awake standing, 16) walking duration in hours, 17) % of time awake walking, 18) absolute time upright (a combination of standing and walking) in hours, 19) % of time awake upright and 20) number of sit to stand transitions (when a sitting event was followed by a standing event).

### Statistical analysis

Data was reported in the main analysis as a progression from the first day of measurement to the last. To control for any weekday/weekend possible effect on the data, a parallel analysis was made with the data reordered by the weekdays (Monday to Sunday), where both time blocks included weekends, and the averages were calculated to control any weekday/weekend possible effect on the data.

Statistical analysis was conducted using IBM SPSS Statistics software (2021 Version 28.0. IBM Corp.: Armonk, NY, USA). Each possible endpoint is described overall, separated by capacity to walk (see below) and by time-block. Distribution of values for each endpoint were tested for normality using a Kolmogorov–Smirnov statistic and compared by time-blocks using a repeated measures t-test or the Friedman test, depending on whether normality was met. A more accurate study on the differences in sensitivity to movements between the two time-blocks considered (0:00 AM -00:00 AM vs- 12:00 PM -12:00 PM) was carried out considering variations in average changes through the five days of measurement. First, considering only the matching days of the week (*n* = 74) and second considering all available days as sequential data gathering (*n* = 95). A generalized linear model with mixed effects was used, including a random effect for individual variations. These analyses take into account that each individual scores around a personal baseline along time observations.

To assess the variability of the weekdays and weekends, the days according to the calendar were taken and entered into the random effects model (not reported). In order to assess the minimum number of days wearing the activPAL3 was needed to attain maximum reliability, a one-factor model estimating the reliability for each measurement day loading in the same latent factor (congeneric model) was used [54]. This model was fitted separately for each one of the variables considered. The model assumed that every subject could have had a different random mean and every day had a different error variance. An improvement in the model fit was considered according to a statistically significant increase in the comparative chi-square goodness of fit statistic (p < 0.05) and an increase in the goodness of fit statistics as the Comparative fit index (CFI), and the Tucker–Lewis index (TLI) larger than 0.05, while the root mean square error of approximation (RMSEA) should stay near 0.05. The reliability of the measurements was assessed with the Cronbach's Alpha and the ICC. The threshold of reliability was set at > 0.80 indicating high reliability, and 0.90 indicating very high reliability [55]. Models not including random effects were also considered (not reported).

Regarding the minimum number of days advisable to ensure an adequate reliability for each time block, data was assessed using the ICC. A threshold reliability was set between 0.80 and 0.89 indicating high reliability, and ≥ 0.90 indicating very high reliability, and the minimum number of days needed was found. A repeated measures t-test was used to assess differences in the ICC between the first two days. Further validation evidence were searched by analysing separately individuals without capacity to stand and walk and individuals with no limitation. Average measurement reliability for the two first days was estimated using ICC (assuming a parallel measurements model with a random effect for individuals) for those residents able to stand and walk and those unable to do so, and the 95% confidence interval was also computed. Additional measurement days were then added until the values of 0.8 and 0.9 for the ICC were reached (corresponding to good and very good reliability thresholds, respectively).

## Results

### Sample characteristics

**Fig. 1:**
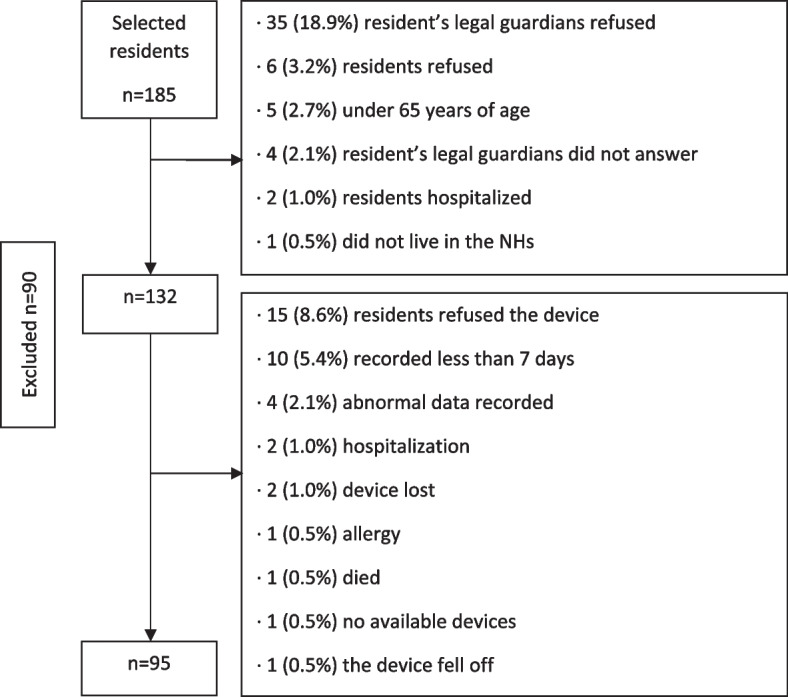
Flowchart of the sampling process of NH residents with the activPAL3

The final sample included 95 residents, mostly women (81.1%), with a mean age of 85.8 ± 7.2 years old. Figure 1 shows the flow chart of the sampling process.

Up to 81.8% of residents had moderate to severe restrictions for all activities of daily living, physical performance disability (57.9%), moderate to severe cognitive impairment (62.2%) and urinary incontinence (69.5%). Regarding sample stratification by their capacity to stand and walk, no discrepancies were found between the answers from the NH staff and the results of the SPPB. The participants able to stand and walk were slightly more than half of the sample (57.9%), compared with those unable (42.1%). The group able to stand and walk had fewer activities of daily living limitations, lower cognitive impairment and were more continent than the group unable to stand and walk. Regarding the SB and ATMB variables, both groups spent more time in SB than standing or stepping. As expected, the able group walked more, and sat less, than the unable group, who spent almost all their waking time in SB. Table 1 shows detailed information about the socio-demographic, physical and psycho-cognitive characteristics of the sample.

**Table 1:** Socio-demographic, physical and psycho-cognitive characteristics of the residents

**Variable**	**Total (*n* = 95)**	**Capacity to stand and walk groups**	
		**Able (*n* = 55)**	**Unable(*n* = 40)**
Age (mean, SD)	85.85 ± 7.24	84.82 ± 6.09	87.28 ± 8.44
Sex n, (%)			
Male	18 (19.0%)	12 (21.8%)	6 (15.0%)
Female	77 (81.1%)	43 (78.2%)	34 (85.0%)
ADL limitations (Barthel) n, (%)			
Independent	5 (5.3%)	5 (9.1%)	0
Slight dependency	16 (16.8%)	16 (29.1%)	0
Moderate dependency	36 (37.9%)	31 (56.4%)	5 (12.5%)
Severe dependency	38 (40.0%)	3 (5.5%)	35 (87.5%)
Cognitive state (Pfeiffer) n, (%)			
Normal mental function	20 (21.1%)	18 (32.7%)	2 (5.0%)
Mild cognitive impairment	14 (14.7%)	12 (21.8%)	2 (5.0%)
Moderate cognitive impairment	20 (21.1%)	12 (21.8%)	8 (20.0%)
Severe cognitive impairment	39 (41.1%)	12 (21.8%)	27 (67.5%)
Unknown	2 (2.1%)	1 (1.8%)	1 (2.5%)
Urinary Incontinence (MDS) n, (%)			
Always continent	29 (30.5%)	27 (49.1%)	2 (5.0%)
Occasionally incontinent	30 (31.6%)	19 (34.5%)	11 (27.5%)
Frequently incontinent	13 (13.7%)	2 (3.6%)	11 (27.5%)
Always incontinent	23 (24.2%)	7 (12.7%)	16 (40.0%)
Physical performance (SPPB) n, (%)			
Robustness	5 (5.3%)	5 (9.1%)	0
Prefrailty	11 (11.6%)	11 (20.0%)	0
Frailty	22 (23.2%)	22 (40.0%)	0
Disability	55 (57.9%)	15 (27.3%)	40 (100.0%)
Unknown	2 (2.1%)	2 (3.6%)	0
Score (mean, SD)	3.10 ± 3.40	5.43 ± 2.7	0
SB and ATMB variables (mean, SD)			
Hours awake (h)	12.49 ± 1.81	13.39 ± 1.39	11.25 ± 1.58
Standing duration (h)	1.80 ± 2.05	2.89 ± 2.04	0.29 ± 0.66
% time awake standing	13.4 ± 14.5	21.3 ± 14.1	2.6 ± 5.3
Walking duration (h)	0.42 ± 0.55	0.72 ± 0.56	0.01 ± 0.04
% time awake walking	3.2 ± 4.5	5.5 ± 4.7	0.1 ± 0.2
Absolute time upright (h)	2.22 ± 2.41	3.61 ± 2.26	0.31 ± 0.70
% time awake upright (h)	16.70 ± 17.51	26.85 ± 16.19	0.80 ± 5.54
Number of sit to stand transitions	24.14 ± 19.91	35.73 ± 18.31	8.20 ± 6.41
Absolute time in SB (h)	10.26 ± 2.10	9.77 ± 2.29	10.93 ± 1.60
% time awake in SB	83.3 ± 17.5	73.1 ± 16.2	97.2 ± 5.5
Number of SB bouts < 30 min (h)	19.22 ± 18.92	29.51 ± 18.57	5.08 ± 5.57
Absolute time spent in bouts < 30 min (h)	1.74 ± 1.62	2.75 ± 1.40	0.35 ± 0.47
% time awake in bouts < 30 min	13.2 ± 12.0	20.5 ± 10.5	3.2 ± 4.0
Number of SB bouts between 30–60 min	2.35 ± 2.23	3.84 ± 1.70	0.30 ± 0.75
Absolute time spent in bouts between 30–60 min (h)	1.65 ± 1.53	2.65 ± 1.22	0.28 ± 0.51
% time awake in bouts between 30–60 min	12.4 ± 11.0	19.6 ± 8.4	2.6 ± 4.6
Number of SB bouts > 60 min	2.58 ± 1.06	2.44 ± 1.18	2.78 ± 0.86
Absolute time spent in bouts > 60 min (h)	6.86 ± 3.86	4.36 ± 2.79	10.30 ± 2.05
% time awake in bouts > 60 min	57.7 ± 34.5	33.1 ± 22.2	91.5 ± 12.6
Average duration of SB bouts (min)	73.90 ± 94.11	24.46 ± 29.57	141.87 ± 109.31

*SD* Standard deviation, *MDS* Minimum Data Set 3.0, *SPPB* Short Physical Performance Battery, *SB* Sedentary behaviour, *ATMB* Awake time movement behaviour, *h* Hours, *min* Minutes, *%* Percentage

### Time block eligibility

There were no statistically significant differences between time blocks (midday time block: 12:00 PM to 12:00 PM; midnight time block: 00:00 AM to 00:00 AM) in ten of the analysed variables. Statistically significant differences were found in the other ten variables: number of sit to stand transitions and SB time distribution between time blocks; specifically, in the number, length, percentage of duration of the SB bouts and in the average duration of SB bouts (Table 2).

**Table 2:** Results from SB and ATMB variables processed by time blocks

**Variables**	**Midnight** **(median ± IQR)**	**Midday** **(median ± IQR)**	**Normality** **KS**	**Z difference**	***P* value**
Hours awake (h)	12.49 ± 1.81^b^	12.46 ± 1.80^b^	.200	-.028^a^	.438
Standing duration (h)	0.87 ± 2.66	0.85 ± 2.62	< .001	-.279	.780
% time awake standing	8.03 ± 20.61	7.59 ± 20.16	< .001	-.102	.919
Walking duration (h)	0.26 ± 0.73	0.26 ± 0.65	< .001	-.072	.943
% time awake walking	2.09 ± 4.94	2.07 ± 4.88	< .001	-.158	.874
Absolute time upright (h)	1.28 ± 3.45	2.29 ± 3.51	< .001	-.294	.769
% time awake upright	11.49 ± 27.04	11.23 ± 27.62	< .001	-.056	.955
Number of sit to stand transitions	21.16 ± 29.50	21.33 ± 29.17	< .001	-5.368	< .001*
Absolute time in SB (h)	10.63 ± 2.41	10.61 ± 2.29	.001	-.795	.427
% time awake in SB	88.50 ± 27.04	88.76 ± 27.62	< .001	-.056	.955
Number of SB bouts < 30 min	16.16 ± 25.33	15.66 ± 26.0	< .001	-3.932	< .001*
Absolute time spent in bouts < 30 min (h)	1.43 ± 2.63	1.52 ± 2.70	< .001	-6.658	< .001*
% time awake in bouts < 30 min	10.57 ± 18.74	12.54 ± 19.23	< .001	-6.658	< .001*
Number of SB bouts between 30–60 min	2.33 ± 4.0	2.33 ± 4.00	< .001	-.858	.391
Absolute time spent in bouts between 30–60 min (h)	1.62 ± 2.84	1.55 ± 2.75	< .001	-2.704	.007*
% time awake in bouts between 30–60 min	11.99 ± 20.71	11.92 ± 20.50	< .001	-2.993	.003*
Number of SB bouts > 60 min	2.33 ± 1.33	2.83 ± 1.50	< .001	-4.041	< .001*
Absolute time spent in bouts > 60 min (h)	6.47 ± 7.72	6.35 ± 7.63	< .001	-5.473	< .001*
% of time awake in bouts > 60 min	54.39 ± 72.61	52.18 ± 71.79	< .001	-6.161	< .001*
Average duration of SB bouts (min)	29.14 ± 99.10	29.53 ± 80.89	< .001	-6.832	< .001*

*KS* Kolmogorov–Smirnov, *SB* Sedentary behaviour, *ATMB* Awake time movement behaviour, *Midnight* Midnight time block, *Midday* Midday time block, *IQR* Interquartile range, *h* Hours, *min* Minutes, *%* Percentage

^a^ Two-related means T-test mean difference

^b^ Mean ± standard deviation

^*^ Statistically significant (*p* < 0.05)

The results of the sensitivity of the measurements through the dispersion frequencies (Table 3) showed that for 10 variables the midnight time block had more sensitivity (wider range) than the midday time block. The midday time block showed more sensitivity on their measurements for nine variables. Finally, for the data of 1 variable, both time blocks had the same sensitivity. These results showed that the midnight time block had a slightly more measurement sensitivity of the variables than midday time block.

**Table 3:** Sensitivity of the measurements results through dispersion frequencies of the data from the two-time blocks

	**Day 1**		**Day 2**		**Day 3**		**Day 4**		**Day 5**		**Day 6**	
	**Min**	**Max**	**Min**	**Max**	**Min**	**Max**	**Min**	**Max**	**Min**	**Max**	**Min**	**Max**
Hours awake												
Midday	5.73	16.88	7.03	18.33	7.12	16.80	6.85	16.60	2.00	16.00	7.63	18.28
**Midnight**	5.87	21.32	6.68	16.72	5.33	19.28	6.07	16.55	6.97	17.07	4.58	16.70
Standing duration (h)												
Midday	0	11.86	0	11.04	0	10.70	0	9.49	0	9.18	0	9.19
**Midnight**	0	11.55	0	10.77	0	11.15	0	10.54	0	8.41	0	9.80
% time awake standing												
Midday	0.0	82.57	0.0	76.93	0	75.12	0	65.89	0	62.71	0	61.73
**Midnight**	0.0	79.64	0.0	74.98	0	78.06	0	73.94	0	54.46	0	65.84
Walking duration (h)												
Midday	0	2.53	0	2.74	0	2.48	0	2.94	0	2.59	0	3.38
**Midnight**	0	3.22	0	2.99	0	2.32	0	2.77	0	2.61	0	3.39
% time awake walking												
Midday	0	25.0	0	21.90	0	18.59	0	24.10	0	21.70	0	29.90
**Midnight**	0	27.68	0	24.19	0	17.65	0	20.73	0	23.22	0	30.24
Absolute time upright (h)												
Midday	0	12.45	0	11.54	0	11.19	0	9.92	0	9.67	0	9.68
**Midnight**	0	12.12	0	11.29	0	11.60	0	11.00	0	9.23	0	10.31
% time awake upright												
Midday	0	86.49	0	80.42	0	78.50	0	75.24	0	66.06	0	66.89
**Midnight**	0	83.59	0	78.59	0	81.22	0	77.20	0	66.88	0	69.30
Number of it to stand transitions												
**Midday**	2	155	2	118	2	132	2	133	2	93	2	122
Midnight	1	141	1	130	1	116	1	159	1	102	1	104
Absolute time in hours of SB (h)												
**Midday**	1.94	15.89	2.81	16.57	3.06	15.31	2.80	15.50	1.96	14.88	3.75	15.85
Midnight	2.38	15.63	3.08	15.61	2.68	16.97	3.14	15.45	3.69	14.94	4.03	15.60
% time awake in SB												
**Midday**	13.51	100	19.58	100	21.50	100	24.76	100	33.94	100	33.11	100
Midnight	16.41	100	21.41	100	18.78	100	22.80	100	33.12	100	30.70	100
Number of SB bouts < 30 min												
**Midday**	0	154	0	117	0	130	0	131	0	92	0	122
Midnight	0	141	0	129	0	113	0	159	0	100	0	103
Absolute time spent in bouts < 30 min (h)												
**Midday**	0	8.34	0	6.24	0	6.59	0	7.32	0	6.70	0	8.20
Midnight	0	6.53	0	6.44	0	6.52	0	6.83	0	6.26	0	6.68
% time awake in bouts < 30 min (h)												
**Midday**	0	58.26	0	45.24	0	50.06	0	57.85	0	56.02	0	56.14
Midnight	0	56.47	0	51.09	0	53.03	0	53.73	0	47.98	0	48.11
Number of SB bouts between 30–60 min												
**Midday**	0	8	0	9	0	10	0	10	0	10	0	8
Midnight	0	11	0	7	0	8	0	9	0	11	0	8
Absolute time spent in bouts 30–60 min												
**Midday**	0	6.07	0	6.33	0	7.03	0	7.02	0	6.88	0	6.31
Midnight	0	7.18	0	4.89	0	6.07	0	6.32	0	8.05	0	5.70
% time awake in bouts between 30–60 min												
**Midday**	0	43.33	0	48.33	0	44.90	0	52.55	0	46.19	0	45.58
Midnight	0	49.12	0	47.45	0	41.03	0	40.99	0	47.15	0	41.99
Number of SB bouts between > 60 min												
Midday	0	6	0	7	0	6	0	6	0	5	0	6
**Midnight**	0	7	0	6	0	6	0	7	0	6	0	6
Absolute time spent in bouts > 60 min												
Midday	0	14.23	0	14.0	0	13.87	0	14.17	0	13.23	0	13.46
**Midnight**	0	13.47	0	14.1	0	14.12	0	13.36	0	14.55	0	14.04
% time awake in bouts between > 60 min												
**Midday**	0	100	0	100	0	100	0	100	0	100	0	100
**Midnight**	0	100	0	100	0	100	0	100	0	100	0	100
Average duration of SB bouts in minutes												
Midday	2.69	330.0	3.05	420.0	3.55	416.0	2.90	366.0	4.38	360.0	2.57	360.5
**Midnight**	2.75	660.0	2.72	699.0	4.18	847.0	2.58	720.0	3.80	719.0	3.33	722.0

Block time in bold indicates the more sensitive time block for each outcome measure

*Min* Minimum value of data dispersion, *Max* Maximum value of data dispersion, *Midnight* Midnight time block, *Midday* Midday time block, *h* Hours, *min* Minutes, *%* Percentage

Only two variables (number of SB bouts > 60 min and the average duration of SB bouts in minutes) had statistically significant differences between time blocks averages. The rest of the variables showed no statistically significant differences between time blocks averages. None of the variables showed statistically significant differences between the six days for each time block (Table 4).

**Table 4:** Fixed effects ANOVA results between time blocks

**Variable**	**F**	***P* value**
Awaking hours (h)		
Between time blocks	.055	.814
Between days	.644	.666
Standing duration (h)		
Between time blocks	.008	.930
Between days	.411	.841
% time awake standing		
Between time blocks	.001	.972
Between days	.419	.835
Walking duration (h)		
Between time blocks	.010	.921
Between days	.113	.990
% time awake walking		
Between time blocks	.001	.972
Between days	.419	.835
Absolute time upright (h)		
Between time blocks	.010	.922
Between days	.311	.906
% time awake upright		
Between time blocks	.002	.968
Between days	.294	.916
Number of sit to stand transitions		
Between time blocks	.237	.627
Between days	.263	.933
Absolute time in SB (h)		
Between time blocks	.007	.933
Between days	.356	.878
% time awake in SB		
Between time blocks	.002	.968
Between days	.294	.916
Number of SB bouts < 30 min		
Between time blocks	.088	.766
Between days	.283	.922
Absolute time spent in bouts < 30 min (h)		
Between time blocks	1.015	.314
Between days	.443	.819
% time awake in bouts between < 30 min (h)		
Between time blocks	1.445	.230
Between days	.419	.835
Number of SB bouts between 30–60 min		
Between time blocks	.326	.568
Between days	.715	.612
Absolute time spent in bouts 30–60 min		
Between time blocks	.385	.535
Between days	.714	.613
% time awake in bouts between 30–60 min		
Between time blocks	.556	.456
Between days	.767	.574
Number of SB bouts between > 60 min		
Between time blocks	3.926	.048*
Between days	.497	.779
Absolute time spent in bouts > 60 min		
Between time blocks	.645	.422
Between days	.184	.969
% time awake in bouts between > 60 min		
Between time blocks	.515	.473
Between days	.120	.988
Average duration of SB bouts (min)		
Between time blocks	7.867	.005*
Between days	.471	.798

*F* F statistic, *h* Hours, *min* Minutes, *%* Percentage

^*^ Statistically significant (*p* < 0.05)

### Reliability of the time blocks data and number of days required

Variability of the weekdays and weekends showed small but significant differences in the day effect between Monday and Sunday, due to variability among individuals (results not reported). For the single first day measure, a very high reliability (ICC > 0.90) was reached in six variables for the midnight blocks and four from the midday block, and a high reliability (ICC > 0.80) was reached in five variables in the midnight block (Table 5). Even although some measures had a good reliability from a single day, they did not always show a stable average over time. Considering the first two days, a very high reliability was reached in ten variables for the midnight blocks and five from the midday block, and a high reliability in three variables in the midnight block. However, for one variable in the midday block reliability was less than an ICC 0.80. About two thirds of the variables had higher values of reliability for the midnight time block than for the midday time block. When considering only the time block with higher reliability for each variable, no statistically significant differences between the two first days were found. Across all the variables, a minimum of four days was needed to achieve an ICC of 0.80, and more than six days for an ICC of 0.90.

**Table 5:** The minimum days reliability between the time blocks

**Variable**	**Time block**	**First day**	**First two days**		**Minimum days to achieve ICC of 0.80**	**Minimum days to achieve ICC of 0.90**
		**ICC**	**ICC**	***P value***		
Awaking hours (h)	Midday	.754	.860	.311	2	3
	Midnight	.747	.852	.511	2	3
Standing duration (h)	Midday	.956	.978	.294	1	1
	Midnight	.929	.963	.550	1	1
% time awake standing	Midday	.942	.970	.260	1	1
	Midnight	.931	.964	.945	1	1
Walking duration (h)	Midday	.910	.953	.480	1	1
	Midnight	.925	.961	.556	1	1
% time awake walking	Midday	.906	.951	.377	1	1
	Midnight	.908	.952	.749	1	1
Absolute time upright (h)	Midday	.963	.981	.451	1	1
	Midnight	.944	.971	.462	1	1
% time awake upright	Midday	.956	.978	.418	1	1
	Midnight	.948	.973	.862	1	1
Number of sit to stand transitions	Midday	.918	.957	.073	1	1
	Midnight	.923	.960	.872	1	1
Absolute time in SB (h)	Midday	.797	.887	.198	2	3
	Midnight	.821	.901	.843	1	2
% time awake in SB	Midday	.956	.978	.418	1	1
	Midnight	.973	.973	.862	1	1
Number of SB bouts < 30 min	Midday	.908	.952	.169	1	2
	Midnight	.916	.956	1.000	1	2
Absolute time spent in bouts < 30 min (h)	Midday	.864	.927	.696	1	2
	Midnight	.887	.940	.631	1	2
% time awake in bouts < 30 min	Midday	.829	.906	.644	1	2
	Midnight	.852	.920	.575	1	2
Number of SB bouts between 30–60 min	Midday	.695	*.820*	.042*	*2*	*4*
	Midnight	.785	.880	.268	2	3
Absolute time spent in bouts 30–60 min	Midday	.707	.828	.037*	*2*	*4*
	Midnight	.787	.881	.210	2	3
% time awake in bouts between 30–60 min	Midday	.659	*.795*	.040*	*3*	4
	Midnight	.761	.864	.435	2	3
Number of SB bouts between > 60 min	Midday	.487	.655	.714	4	> 6
	Midnight	.457	.628	.777	4	> 6
Absolute time spent in bouts > 60 min	Midday	.865	.928	.613	1	2
	Midnight	.892	.943	.717	1	2
% of time awake in bouts between > 60 min	Midday	.918	.957	.163	1	1
	Midnight	.926	.961	.799	1	1
Average duration of SB bouts (min)	Midday	.774	.873	.060	2	3
	Midnight	.870	.930	.318	1	2

*ICC* Intra-class correlation coefficient, *Midnight* Midnight time block, *Midday* Midday time block, *h* Hours, *min* Minutes, *%* Percentage

^*^ Statistically significant (*p* < 0.05)

### Stratification of the sample and number of days required

Participants were grouped by mobility to explore the minimum number of days required within the midnight time block. The midnight time block variables did not have any statistically significant differences between the days but did have higher reliability and showed higher measurement sensitivity than the midday block. Within the more capable group, between 2 and 3 days of measurement was required to reach a reliability of an ICC 0.80. However, for the less capable group 2–6 days was needed to reach a reliability of 0.80. Finally, to reach a reliability ≥ 0.90, both groups needed more than six days. Table 6 shows more information about the minimum days required.

**Table 6:** The minimum days required for reliable SB and ATMB results stratified by their stand and walk capacity

**Variable**	**Stand and walk capacity**	**First 2 days**	**Minimum days to achieve ICC of 0.80**	**Minimum days to achieve ICC of 0.90**
		**ICC (95% CI)**		
Awaking hours (h)	Able	.782 (.656–0.774)	3	5
	Unable	.821 (.662-.906)	2	4
Standing duration (h)	Able	.941 (.898-.965)	2	2
	Unable	.940 (.887-.968)	2	2
% time awake standing	Able	.945 (.905-.968)	2	2
	Unable	.904 (.819-.949)	2	2
Walking duration (h)	Able	.936 (.890-.962)	2	2
	Unable	.953 (.911-.975)	2	2
% time awake walking	Able	.926 (.873-.957)	2	2
	Unable	.914 (.838-.955)	2	2
Absolute time upright (h)	Able	.947 (.909-.969)	2	2
	Unable	.946 (.897-.971)	2	2
% time awake upright	Able	.954 (.921-.973)	2	2
	Unable	.912 (.834-.953)	2	2
Number of sit to stand transitions	Able	.948 (.911-.970)	2	2
	Unable	.704 (.441-.844)	3	6
Absolute time in SB (h)	Able	.918 (.859-.952)	2	2
	Unable	.808 (.637-.898)	2	4
% time awake in SB	Able	.954 (.921-.973)	2	2
	Unable	.912 (.834-.953)	2	2
Number of SB bouts < 30 min	Able	.948 (.910-.969)	2	2
	Unable	.671 (.378-.826)	3	> 6
Absolute time spent in bouts < 30 min (h)	Able	.905 (.838-.945)	2	2
	Unable	.717 (.465-.850)	3	> 6
% time awake in bouts < 30 min	Able	.891 (.814-.937)	2	3
	Unable	.623 (.287-.801)	4	> 6
Number of SB bouts between 30–60 min	Able	.778 (.619-.870)	3	> 6
	Unable	.564 (.176-.769)	5	> 6
Absolute time spent in bouts 30–60 min	Able	.792 (.644-.879)	3	> 6
	Unable	.497 (.049-.734)	5	> 6
% time awake in bouts between 30–60 min	Able	.791 (0.641–0.878)	3	> 6
	Unable	.446 (-0.047–0.707)	6	> 6
Number of SB bouts between > 60 min	Able	.698 (0.483–0.824)	3	> 6
	Unable	.379 (-0.174–0.672)	5	> 6
Absolute time spent in bouts > 60 min	Able	.901 (.830-.942)	2	2
	Unable	.774 (.574-.881)	3	4
% time awake in bouts between > 60 min	Able	.913 (.851-.949)	2	2
	Unable	.759 (.544-.872)	3	5
Average duration of SB bouts (min)	Able	.754 (.578-.857)	3	5
	Unable	.913 (.836-.954)	2	2

*ICC* Intra-class correlation coefficient, *CI* Confidence Interval, *h* Hours, *min* Minutes, *%* Percentage, > *6* more than 6 days

## Discussion

This study aimed to determine the minimum number of days of activPAL3 monitoring required to reliably examine SB and ATMB in NH residents and the best time blocks in which to process the data. Our findings indicate that a minimum of 3 consecutive days wearing the activPAL3 device is required to achieve high reliability for those residents with capacity to stand and walk, and 6 consecutive days for those residents unable to stand and walk across a range of SB and ATMB variables. We also found that processing data from midnight to midnight was more reliable than processing from midday to midday.

The minimum number of days required to adequately measure SB and ATMB in NH residents may be dependent on a number of factors, including the monitor used and outcome measures explored, and the actual levels and variability between individuals in daily patterns of SB and ATMB.

Edwarson et al. noted that a person-oriented approach to analysis of SB and ATMB data can be behaviourally relevant, but that in community-dwelling adults and older adults person-oriented day durations (from one wake time to the next) are not always 24 h long [45]. In other words, people may wake up at different times each day, which may be accentuated in working age adults by differences between week (working) and weekend (non-work) days. Farias-Aguilar et al. found in a community-dwelling working-age adult population, differences in intra-individual variability between weekdays and weekend days, in that there was slightly less variability in SB and activity behaviours on weekends compared to weekdays [56]. However, person-oriented day duration and intra-individual and inter-individual variability between days of the week for NH residents are likely to be different from that of the working adult population. This is because NH residents spend most of their day lying or sitting during their daily activities, particularly related to mobility and feeding [37]. In most NHs the daily routines are set by the institution management and there is a high level of control by staff, shaping residents' daily routines [37, 57]. In line with our findings, Buckley et al. and Airlie et al. found that there was no difference in most outcome measures between weekdays and weekend days in NH residents [58, 59]. Therefore, for this population, it may not be necessary to include a weekend day in the assessment period, and older NH residents are not bound to a typical weekday/weekend week structure. Considering that the daily routines of NH residents are strongly conditioned, we suggest that their person-oriented day durations are likely to approximate to 24 h long (similar wake times each day) without significant differences between weekdays and weekends.

Most recent studies using the activPAL3 device report the choice of a 24-h wear protocol, meaning the monitor is worn continuously including overnight, but there is a lack of consensus on many other protocol decisions such as timing of starting to wear the monitor, what time blocks, and reporting on data processing decisions such as when data is processed [33, 46, 60]. This lack of consistency can potentially lead to discrepancies in data interpretation and make comparisons between studies difficult. For example, Reid et al. measured activity patterns among older adults in residential aged care using the activPAL3, they reported using a 24-h protocol for 7 days, but do not report the starting hour of the monitoring period or if the data of the first and last days were composed from partial days or were whole days [33]. Bootsman et al. also reported, using a 24-h wear protocol, with the activPAL3 in older adults living in residential aged care facilities for five days. They reported that the participant started wearing the monitor during the day, but that measurement only started at midnight, which was to minimize potential differences in movement behaviours during the first few hours of wear [60]. In a study measuring SB of community-dwelling older adults with the activPAL3, Dall et al. reported programming the monitor to start recording immediately with a recording duration of 14 days and that the monitor was then put on the participant at an unspecified later date and time [46]. The protocol specified the devices were taken off from the ninth day of wear onwards at an unspecified time. Data was then processed into midnight-to-midnight time blocks to extract 7 days of data each 24-h long and starting at the same time. Our study supports their choice of midnight time blocks for analysis, as they offer greater sensitivity and showed no statistically significant differences between days. This was perhaps surprising, as the analysis covers the same activity of each individual, and the only difference between time-blocks is that the midnight time block more usually represents a single person-oriented day (e.g. going to sleep before midnight on the day that you woke up on), than a composite from two person-oriented days (midday to sleep time on one day and the wake time to midday of the following day). It is unclear whether analysing a true person-oriented approach (wake time to wake time) would also represent a reliable method, or how these results would translate to a community-dwelling population, where more diversity of the timing of wake and sleep would be likely.

To the best of the authors' knowledge, the study by Reid et al. is the only one that examines the minimum requirements for obtaining reliable estimates in older adults residing in care homes in Australia using the activPAL3 device for measurement [33]. Reid et al. explored three standard outcome measures, and found it would take 5 to 11 days to estimate sitting time, 5 to 10 days for standing time, and 7 to 15 days for stepping time to achieve an ICC of 0.8 to 0.9. In contrast, for those three outcome measures, our study required a minimum of 2 to 4 days. Even across the full range of outcome measures we explored, the minimum days required were 3 for residents with the capacity to stand and walk and 6 for those who were unable to stand and walk. These are considerably shorter than the minimum days required by Reid et al. [33]. Participants in the two studies appear to be reasonably similar, in terms of mean age, 84.2 and 85.8, although some of the potentially more frail NH residents (individuals with pacemakers, behavioural issues, uncommunicable deafness or diagnosed severe dementia) were excluded from Reid’s study, but included in ours. Therefore, the main reason for differences in minimum number of days required between the two studies might be due to different sample sizes (*n* = 31, in Reid et al. vs *n* = 95 in the current study). Indeed, when we calculated the reliability stratified into two walking capacity groups, compared with the entire sample, the reduction in sample size resulted in a loss of heterogeneity, which affected the reliability of the results. Consequently, more days were needed to ensure an ICC of 0.8 or 0.9, to reach more accurate and reliable measure of the variable being studied.

Buckley et al., in 2020, explored the minimum reliable days of device wearing for walking activity in a sample of 257 NH residents in New Zealand, measured using a different device (Axivity AX3 device, worn on the lower back) [58]. Data was recorded across 8 days, and divided into 7 days for analysis using the half days on the first and eighth day of measurement. Although the start time of the days was not reported, this is functionally similar to our midday time block. Buckley et al. focused on walking and assessed volume variables of total walk time, total steps and total number of walking bouts and pattern variables, including mean walking bout length. Results were presented for the whole group, number of days required ranged from 2 to 5 days across all variables, and stratified by level of care, the dementia level of care ranged from 1 to 3 days, the intermediate ranged from 2 to 7 days, and the high level of care ranged from 2 to 6 days. Although the device used and the variables explored were different, our results are in line with their range of minimum days. Also, in line with our findings, the number of days of measurement required for volume-based metrics was lower than those for pattern-based metrics. Buckley et al., also classified their sample in groups according to their level of care, whereas we grouped participants according to their physical capacity to stand and walk. The number of days required for measurement for those who were able to stand and walk in our study [2, 3] was similar to the dementia care group [1–3], whereas the days required for the group who could not mobilise in our study [2–6] was similar to both the intermediate care group [2–7] and the high care group [2–6]. However, when it comes to issues of dementia, level of care does not necessarily equate to ability to mobilise. Indeed, individuals in the high care group showed better cognition status according to the Montreal Cognitive Assessment (MoCA) than both the intermediate and the dementia level of care groups, but had worse physical function assessed using the Time Up and Go (TUG). Also, as a group, those with the dementia level of care had the best physical function. In comparison, our results showed that the more disabled group had both a higher physical and cognitive impairment (68% with severe cognitive impairment) compared with the able to stand and walk group (23% with severe cognitive impairment). This suggests that it is the influence of physical performance capacity, rather than the cognitive status, that determined how many days of measurement are required, potentially because all of the variables assessed in both studies are related to physical performance. Another possible reason why a more physically impaired group might need more days to guarantee the reliability of data is the high homogeneity of their daily routines and their sensitivity to PA. These residents spend almost all of their waking hours in SB and their PA bouts are typically limited to the same daily routine (e.g., toilet, hygiene, or feeding) and depend on the availability of NH staff and their assistance. Therefore, any PA bout outside of their daily routine would become unusual (e.g., if a resident requires assistance due to an incontinence event). This isolated PA bout would be enough to make a large difference in both the volume and pattern of PA between days, thus reducing the reliability of the data. Consequently, more days of assessment may be needed to reach higher reliability values.

In a similar manner, Airlie et al. (2022) determined the minimum number of days of wear and optimal wear time criteria required to assess PA and SB, measured using a different device (ActiGraph wGT3X + worn on the right hip), in a sample of 91 NH residents in the United Kingdom with preserved mental capacity [59]. Data were recorded over the course of 7 days. Although the starting time of recording was not reported, it was mentioned that the first monitoring day was excluded if the monitor was administered after 1 PM. This implies that they did take into consideration the starting time of recording with the device and excluded data from the first monitoring day if it began after 1 PM. Moreover, they used data from half-days, but they did not employ data from half-days with less than 4 h of wear time, similar to our midday time block. However, they did not follow a 24-h protocol, as the NH residents were instructed to remove the device before engaging in any water-based activities. Instead, they utilized a diary log for wear time, where residents were required to report the day, time, and reason if they removed the device. Furthermore, the procedure for completing the activity log was clarified to the staff, who were also requested to provide assistance where necessary. However, the method by which they obtained awake time and excluded night time data is not reported.

The results from Airlie et al. indicated that estimates of accelerometer outcomes as counts per day, counts per minute, PA time in minutes, and SB time in minutes, did not significantly differ by monitoring day (weekdays or weekend) like our results and Buckley’s., and the accelerometer outcomes were equivalent regardless of the employed minimum daily wear time criterion. This suggests that accelerometer outcomes are consistent and reliable in NH population. Additionally, the study examined the impact of the number of monitoring days on the reliability of accelerometer outcomes. Results showed that estimates of counts per minute were equivalent regardless of the number of monitoring days. However, for counts per day and PA time, only estimates based on at least 6 monitoring days were considered equivalent to estimates based on 7 monitoring days. These findings suggest that a 7-day monitoring protocol remains advisable to ensure reliable estimates of PA and SB.

The requirement for a 7-day monitoring protocol can be attributed to the fact that nearly half of the sample consisted of dependent individuals, and the analysis was conducted by considering both dependent and independent residents together. When comparing these results to our findings, the initial analysis of minimum days of reliability did not involve stratifying the sample, leading to the reporting of more than six days required in one variable. However, after stratifying the sample based on their capacity to stand and walk, the more impaired group indicated a greater need for assessment days across multiple variables when compared to those with the ability to stand and walk. These findings further support the previously suggested hypothesis that the influence of physical performance capacity, rather than cognitive status, determines the necessary number of measurement days.

The minimum number of days wearing the activPAL3 has been explored in a few other populations, including asymptomatic female adolescents, working adults, middle age women and adults and older adults receiving hemodialysis. Female adolescents (*n* = 195, with a mean age of 15.7 (SD = 0.9) years old), assessed using a 7-day monitoring period, required a minimum of 12 days to achieve a reliability of ≥ 0.8 for the variables time spent sitting or lying, standing time, light PA (LPA), and moderate-to-vigorous PA (MVPA), while 21 days were necessary for assessing the number of steps [61]. Working adults (*n* = 90, with a mean age of 39.1 (SD = 12.43) years old), assessed using a 7-day monitoring period, required a minimum of 5 days (with at least 1 weekend day included) to achieve a reliability of 0.8 for the variables sitting or lying time, standing time, and stepping time, while transitions to standing required at least 3 days [56]. Middle age women (*n* = 68, with a mean age of 52 (SD = 8) years old), assessed during a 7-day monitoring period, required a minimum of 4 days an ICC of 0.80 for the variables sitting or lying time and LPA and 9 days were needed for an ICC > 0.9 [62]. For adults and older adults receiving hemodialysis (*n* = 70, with a mean age of 55.9 (SD = 15.7) years old), assessed during a 7-day monitoring period, required a minimum of one dialysis day and two non-dialysis days for an ICC of 0.80 for the variables of waking hours, percentage of time spent sitting or lying, percentage of waking time spent standing, the number of transitions to standing per hour, number of steps taken per day, number of steps taken per minute and energy expenditure per minute [63]. In general, as the participants in these studies get older, the number of days required to wear the activPAL gets lower. However, it is likely that it is how age impacts daily routine, and thus intra- and inter-individual variability, that may be important [56, 63]. This is supported by the study of individuals receiving haemodialysis, where it was suggested that days with and without dialysis, which likely had very different patterns of activity were included in the measurement period. Prescott et al.'s also suggested that comorbidities, and lower levels of functional independence, can lead to lower inter- and intra-individual variability [63]. Our findings suggest that variability is affected mainly by the high level of schedule control exercised by NH staff in residents' daily routines and also by the participants' ability to stand and walk. Moreover, in those who are unable to stand and walk, the variability would also be affected by their dependency to the NH staff and their assistance for their daily routines [37, 57].

Our study has several limitations. One limitation of this study is sample size as data collection was stopped in March 2020 due to the covid-19 outbreak. However, the sample size of 95 in this study is larger than, or similar to, other studies exploring minimum number of days of wear [33, 56, 62, 63]. The final sample analysed was 51% of those invited to take part in the study, which may not be representative of all NH residents. Also, the sample is specific only to NHs in Catalonia (Spain), which have their own politics, characteristics and context, and may not be generalisable to NHs elsewhere. The study was based on 7 days of activPAL3 measurement, so we can only report on up until < 6 days of wear. We found a high reliability (ICC > 0.8) for all variables explored within 7 days, but a longer period of assessment would be required to explore the number of days required to attain a very high reliability (ICC > 0.9) for some of the variables, in particular those exploring the pattern of SB. The limited availability of devices in the project meant that assessment was started for residents on different days of the week. Another potential limitation was the inability to use a diary log to document waking times, bedtimes, and napping within the NH population. During the pilot study, the team attempted to instruct the residents on how to complete the diary correctly. However, at the end of the assessment, after seven days of wearing the activPAL device, none of the residents returned their completed diaries to the research team. Subsequently, we sought input from NH staff, but their responses, though provided, were general and inaccurate for all residents (e.g. everyone getting up at 9:00 AM and going to bed at 10:00 PM). Due to the lack of compliance among NH residents, we decided to discontinue the use of diaries and instead focused on analyzing heat maps within the activPAL software to determine waking and bedtime patterns. Because of this decision and the absence of contextual information from the objective measurements provided by the activPAL, we were unable to identify any instances of napping or sleeping during waking hours for NH residents, especially those who were bedridden. In conclusion, we included all data from waking times to bedtimes, acknowledging that in certain cases, the sedentary behavior data might be somewhat inflated. Finally, we excluded variables related to the number of steps taken, due to the risk of the activPAL3 not recording steps in residents with a very low gait speed (< 1.5 km/h) [64]. On the other hand, our study has several strengths. Firstly, the results can help improve compliance with wearable devices among the NH population by reducing the required wearing time, thereby avoiding loss of both the device and data [33, 58]. To our knowledge, this is the first study to assess data eligibility based on time block distribution using the activPAL3 device, and to stratify the sample by residents' capacity to stand and walk in NHs. Additionally, our study provides information on the minimum number of days required for each variable individually, allowing researchers to choose and select variables according to their specific needs. Finally, our study offers pragmatic solutions for researchers working with the gold standard activPAL3 device and those seeking to evaluate interventions aimed at reducing prolonged sedentary bouts and promoting PA among NH residents.

## Conclusions

This study suggests that a minimum of 3 consecutive days wearing the activPAL3 device is required for those NH residents with capacity to stand and walk, to achieve high reliability, and 6 consecutive days for those with the ones who require help to mobilize, to gather reliable data of SB and ATMB variables. The midnight time block as the reference for data processing and removing the half days is recommended, regardless of the activPAL3 recording start time. This information can be useful for future research assessing SB and time-awake movement behaviours in NH residents.

## Data Availability

The datasets used and/or analysed during this study are available from the corresponding author on reasonable request.

## Abbreviations

AM: Ante Meridiem
ATMB: Awaking-time movement behaviours
CFI: Comparative fit index
ICC: Intra-class correlation coefficient
LPA: Light physical activity
MoCA: Montreal Cognitive Assessment
MVPA: Moderate-to-vigorous physical activity
NH: Nursing home
PA: Physical activity
PM: Post Maridiem
RMSEA: Root mean square error of approximation
SB: Sedentary behaviour
SD: Standard deviation
SPPB: Short Performance Physical Battery
TLI: Tucker–Lewis index
TUG: Time Up and Go
